# FRAX486, a PAK inhibitor, overcomes ABCB1-mediated multidrug resistance in breast cancer cells

**DOI:** 10.1590/1414-431X2024e13357

**Published:** 2024-07-01

**Authors:** Meng Zhang, Xiaoqi Zeng, Meiling She, Xingduo Dong, Jun Chen, Qingquan Xiong, Guobin Qiu, Shuyi Yang, Xiangqi Li, Guanghui Ren

**Affiliations:** 1 Department of Thyroid and Breast Surgery, Shenzhen Hospital of Southern Medical University, Shenzhen, Guangdong, China; 2 School of Traditional Chinese Medicine, Southern Medical University, Guangzhou, Guangdong, China; 3 Department of Pharmaceutical Sciences, College of Pharmacy and Health Sciences, St. John's University, New York, USA; 4 Department of Breast Surgery, The Second Affiliated Hospital of Shandong First Medical University, Tai'an, Shandong, China

**Keywords:** FRAX486, p21-activated kinase (PAK) inhibitor, Multidrug resistance, ABC transporter, ABCB1

## Abstract

The overexpression of P-glycoprotein (P-gp/ABCB1) is a leading cause of multidrug resistance (MDR). Hence, it is crucial to discover effective pharmaceuticals that counteract ABCB1-mediated multidrug resistance. FRAX486 is a p21-activated kinase (PAK) inhibitor. The objective of this study was to investigate whether FRAX486 can reverse ABCB1-mediated multidrug resistance, while also exploring its mechanism of action. The CCK8 assay demonstrated that FRAX486 significantly reversed ABCB1-mediated multidrug resistance. Furthermore, western blotting and immunofluorescence experiments revealed that FRAX486 had no impact on expression level and intracellular localization of ABCB1. Notably, FRAX486 was found to enhance intracellular drug accumulation and reduce efflux, resulting in the reversal of multidrug resistance. Docking analysis also indicated a strong affinity between FRAX486 and ABCB1. This study highlights the ability of FRAX486 to reverse ABCB1-mediated multidrug resistance and provides valuable insights for its clinical application.

## Introduction

The development of multidrug resistance (MDR) in malignant tumor cells is a significant obstacle in the treatment of malignancies (1). MDR refers to the capacity of cancer cells to resist the effects of various drug structures and mechanisms of action (2). MDR is caused by multiple mechanisms, including increased drug efflux from cells (3), inhibition of apoptosis (programmed cell death) (4), and enhanced DNA repair (5). Abnormal expression of ATP-binding cassette (ABC) transporter proteins is a significant contributor to the development of MDR (6).

ABC transporters are a family of proteins that transport medications out of cells by utilizing energy from ATP hydrolysis (7,8). By doing so, they reduce the concentration of drugs within the cells, thereby diminishing their effectiveness and resulting in drug resistance. There are 49 types of ABC transporters in humans, 16 of which are implicated in MDR in cancer cells (9-11). ABCB1 (P-glycoprotein), ABCG2 (breast cancer resistance protein), and ABCC1 (multidrug resistance protein) are the primary ABC transporters linked to MDR (12-14).

These ABC transporters are prevalent in both hematologic and solid tumors, and their abnormal activity can interfere with the absorption and distribution of chemotherapeutic agents in cancer patients (15-17). This can ultimately result in the failure of chemotherapy. ABCB1, in particular, is expressed in a variety of normal cells and has the ability to pump out a wide variety of chemotherapy drugs, including paclitaxel, vinca alkaloids, and anthracyclines, thereby enhancing drug resistance in tumor cells (18-20). In order to overcome MDR in cancer treatment, it is crucial to identify agents that can reverse drug resistance (21).

p21-activated kinase (PAK) is a family of serine/threonine protein kinases that play essential roles in various cellular processes (22,23), including cell proliferation, survival, migration, and cytoskeletal remodeling (24-26). PAKs have been linked to a variety of diseases, including cancer (27), neurological disorders (28), and cardiovascular diseases (29), due to their involvement in numerous cellular processes. PAK inhibitors are being studied as potential therapeutic agents for the treatment of cancer, as inhibiting PAK activity may inhibit the growth, migration, and invasion of cancer cells (27). FRAX486 is a PAK inhibitor (30), and no association between PAK inhibitors and ABCB1 has been reported to date.

This study intended to elucidate the potential of FRAX486 as a reversal agent for multidrug resistance mediated by ABCB1, as well as its therapeutic potential in overcoming drug resistance and improving cancer patient treatment outcomes.

## Material and Methods

### Chemical compounds

We purchased FRAX486, paclitaxel, doxorubicin, cisplatin, and verapamil from MedChemExpress (USA). DMEM and FBS were acquired from Gibco, Invitrogen (USA). The monoclonal antibodies against ABCB1 and Alexa Fluor 488 conjugated antibody from Sigma-Aldrich (USA) are widely used and well-established antibodies for detecting and analyzing ABCB1 protein expression.

### Cell lines and cell culture

The multidrug-resistant ABCB1-overexpressing cell line MCF7/ADR was obtained by inducing the MCF7 cells with doxorubicin, purchased from Guangzhou Xinyuan Technology Co., Ltd. (China). HEK293 cells were transfected with either an empty pcDNA3.1 vector or an ABCB1 full-length gene plasmid in order to generate the HEK293/PCDNA3.1 and HEK293/ABCB1 cell lines. All cell lines were grown in a 10% FBS-supplemented DMEM medium.

### CCK8 assay

The cytotoxicity and reversal assays were conducted following a previously established procedure (31). In the cytotoxicity study, cells were seeded and allowed to adhere in 96-well plates. After 12 h, various FRAX486 concentrations were added to the wells. Following this, the cells were incubated for an additional 72 h. Using an enzyme-labeling technique, the absorbance of the cells was determined.

Cells were distributed into 96-well plates for the reversal experiment and allowed to adhere for 12 h. Cells were then exposed to varying concentrations of 1, 0.3, and 0.1 μM of FRAX486 for a period of 2 h, then to 3 μM concentrations of paclitaxel, doxorubicin, and cisplatin. The positive control consisted of the ABCB1 inhibitor verapamil, while the negative control consisted of cisplatin, which lacked ABCB1 substrate properties. Using an enzyme-labeling technique, the absorbance values of the cells were measured after an additional 72 h of incubation.

### Western blotting

Based on a previous study (32), MCF7/ADR cells were treated with or without 1 μM FRAX486 for 0, 24, 48, and 72 h. The total protein content of cells was extracted using lysis buffer (RIPA lysis buffer; MedChemExpress, USA). Using a PierceTM BCA Protein Assay Kit (Thermo Scientific, USA), protein concentrations in cell lysates were measured to ensure equal loading. After 2 h of non-fat milk blocking, 1:1000 dilutions of ABCB1 or GAPDH primary antibody were incubated at 4°C. The next day, after 2 h of room temperature incubation, HRP-labeled secondary antibody (1:1000) was detected by electrochemiluminescence. ImageJ software (NIH, USA) was used to analyze the band density of photographs.

### Immunofluorescence assay

The immunofluorescence assay was conducted according to a specified methodology (33). Following the administration of FRAX486, cells were fixed for 15 min using a 4% formaldehyde solution. Subsequently, cells were subjected to an overnight treatment with primary antibodies at a temperature of 4°C. Following the incubation period, cells were exposed to secondary antibodies under ambient conditions for a duration of 60 min. Cells were treated with DAPI, a fluorescent dye specific for DNA, for 15 min at ambient temperature. The samples were photographed using an EVOS FL fluorescence microscope (Life Technologies Corporation, USA).

### ATPase assay

As previously reported (34), the vanadate-sensitive ATPase activity of ABCB1 in membrane vesicles from High Five insect cells was assessed using the SB-MDR1-Hi5-PREDEASY-ATPase kit obtained from Sigma Chemical Co. (USA). Membrane vesicles (10 μg) were incubated in ATPase assay buffer containing MES, KCl, sodium azide, EGTA, DTT, ouabain, and MgCl_2_, with or without vanadate, at 37°C. They were then incubated with FRAX486 at various concentrations for 3 min at 37°C. A 5-mM solution of Mg-ATP was used to initiate the ATPase reaction in a 100-μL volume for 20 min at 37°C, followed by the addition of 100 μL of a 5%-SDS solution to halt the process. After incubation at 37°C for 40 min, luminescence signals of Pi were measured.

### Doxorubicin accumulation and efflux assay

The accumulation and efflux assay was performed with a FACSort flow cytometer (BD Biosciences, USA) and analyzed with FlowJo software V10 (https://www.flowjo.com). Briefly, MCF7 and MCF7/ADR cells were separated into treatment-specific groups of 2×10^5^ cells each. Two groups were used for culturing cells with verapamil or FRAX486. To each treatment group, 5 μmol/L of fluorescent substrate doxorubicin was administered, either with or without verapamil or FRAX486. Prior to analysis, cells were centrifuged at 500 *g* for 10 min at 4°C and resuspended in 300 μL PBS with 0.5% BSA.

### Molecular docking

As previously reported (35), FRAX486 was docked with the human ABCB1 model. The RCSB Protein Data Bank provided the human ABCB1 protein model (7A69), and PubChem provided FRAX486's molecular structure. Docking was computed with Maestro v11.1 (Schrodinger LLC, USA). After developing the receptor/ligand, Glide XP docking and default induced-fit docking were done.

### Statistical analysis

The experiments were conducted a minimum of three times. The data were analyzed using GraphPad Prism 9.0 software (USA) with one-way ANOVA. All data are reported as means±SD. A significance level of P*<*0.05 was deemed to be statistically significant.

## Results

### FRAX486 restored the sensitivity of chemotherapeutic drugs in ABCB1 overexpression cells

**Figure 1:**
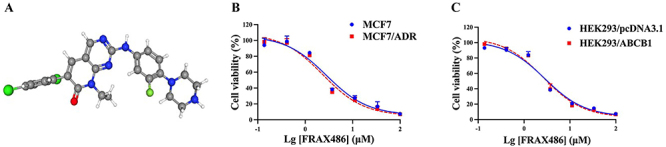
Cytotoxicity of FRAX486 in parental and drug-resistant cells. **A**, Chemical structure of FRAX486. Cell viability curves for (**B**) MCF7 and MCF7/ADR cells and (**C**) HEK293/pcDNA3.1 and HEK293/ABCB1 cells. Data are reported as means±SD for three independent assays (ANOVA).

Figure 1A depicts the chemical structure of FRAX486. To define a suitable concentration of FRAX486, we conducted the CCK8 assay to assess its cytotoxic effects on various cell types. As shown in Figure 1B and C, survival rates of cells treated with 1 μM FRAX486 for 72 h were greater than 80%.

The positive control consisted of the ABCB1 inhibitor verapamil, while the negative control consisted of cisplatin, which lacked ABCB1 substrate properties. Table 1 shows that ABCB1-overexpressing MCF7/ADR cells were more resistant to paclitaxel and doxorubicin than their progenitor MCF7 cells. However, the half maximal concentration (IC_50_) of MCF7/ADR cells decreased as a function of FRAX486 concentration in MCF7/ADR cells. In addition, we assessed the reversal effect of FRAX486 in HEK293/ABCB1 cells transfected with the ABCB1 gene. As shown in Table 1, HEK293/ABCB1 cells treated with 1 μM FRAX486 significantly overcame paclitaxel and doxorubicin resistance. In addition, FRAX486 concentration-dependently enhanced the sensitivity of resistant cells to paclitaxel and doxorubicin. These results demonstrated the efficacy of FRAX486 in reversing multidrug resistance caused by ABCB1.

**Table 1:** Cytotoxicity of FRAX486 in parental and ABCB1-overexpressing cells.

Cell lines	RF (IC_50_, μm)			
	MCF7	MCF7/ADR	HEK293/pcDNA 3.1	HEK293/ABCB1
Doxorubicin	0.231±0.025 (1.000)	9.483±0.707 (41.058)	0.012±0.000 (1.000)	0.587±0.028 (50.381)
+Verapamil (3 μM)	0.297±0.059 (1.284)	1.281±0.019 (5.545)*	0.014±0.001 (1.212)	0.045±0.004 (3.877)*
+FRAX486 (1 μM)	0.193±0.030 (0.836)	1.631±0.212 (7.063)*	0.010±0.001 (0.891)	0.055±0.002 (4.721)*
+FRAX486 (0.3 μM)	0.232±0.029 (1.004)	4.285±0.539 (18.553)*	0.013±0.000 (1.116)	0.236±0.010 (20.211)*
+FRAX486 (0.1 μM)	0.198±0.039 (0.859)	6.293±0.518 (27.247)*	0.014±0.000 (1.181)	0.432±0.012 (37.061)*
Paclitaxel	0.026±0.001 (1.000)	1.050±0.101 (40.405)	0.029±0.001 (1.000)	1.261±0.088 (42.932)
+Verapamil (3 μM)	0.026±0.001 (1.008)	0.153±0.022 (5.868)*	0.034±0.001 (1.149)	0.133±0.006 (4.517)*
+FRAX486 (1 μM)	0.030±0.005 (1.144)	0.178±0.022 (6.846)*	0.025±0.002 (0.867)	0.211±0.018 (7.197)*
+FRAX486 (0.3 μM)	0.032±0.001 (1.221)	0.441±0.020 (16.949)*	0.024±0.001 (0.805)	0.518±0.021 (17.650)*
+FRAX486 (0.1 μM)	0.032±0.001 (1.240)	0.693±0.045 (26.645)*	0.024±0.002 (0.829)	0.799±0.061 (27.197)*
Cisplatin	4.828±0.402 (1.000)	5.184±0.133 (1.074)	5.337±0.281 (1.000)	5.529±0.193 (1.036)
+Verapamil (3 μM)	5.291±0.375 (1.096)	5.830±0.328 (1.207)	5.361±0.175 (1.004)	5.939±0.220 (1.113)
+FRAX486 (1 μM)	4.831±0.502 (1.001)	5.673±0.616 (1.175)	5.500±0.149 (1.031)	5.768±0.084 (1.081)
+FRAX486 (0.3 μM)	6.149±0.835 (1.273)	5.604±0.526 (1.161)	4.806±0.120 (0.900)	5.586±0.222 (1.047)
+FRAX486 (0.1 μM)	5.231±0.554 (1.083)	6.407±0.603 (1.327)	5.786±0.163 (1.084)	4.799±0.252 (0.899)

Resistance fold (RF) was computed by dividing the half maximal concentration (IC_50_) values of substrates with or without the inhibitor by the IC_50_ of parental cells without inhibitor. Data for IC_50_ are shown as means±SD of at least three independent tests. *P<0.05 *vs* control treatment (verapamil) (ANOVA).

### FRAX486 did not affect ABCB1 protein expression

**Figure 2:**
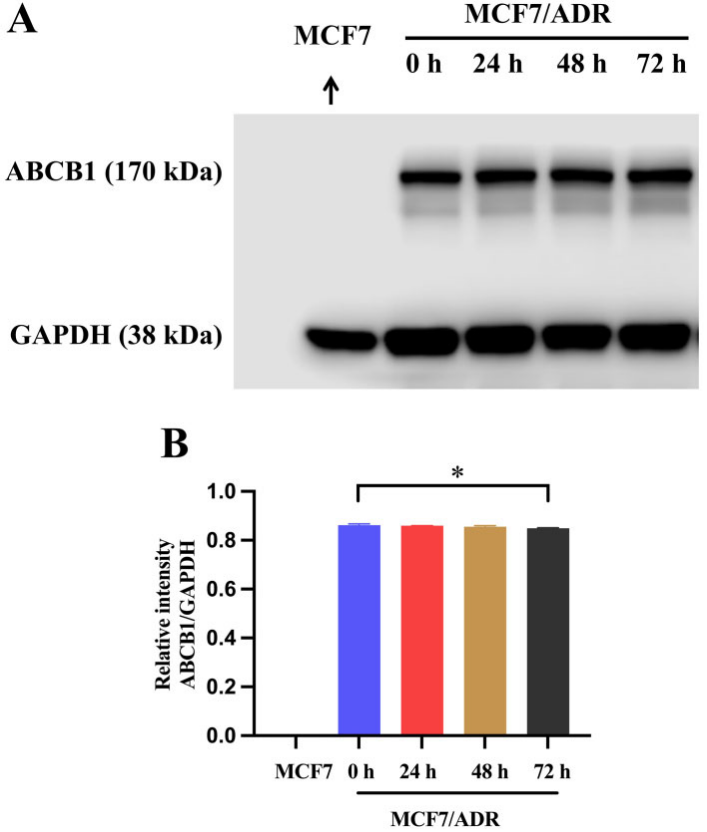
**A**, Effect of FRAX486 on the protein expression of ABCB1 transporters in MCF7/ADR cells incubated with 1 µM of FRAX486 for 0, 24, 48, and 72 h. **B**, Quantitative analysis of protein expression. Data are reported as means±SD, representative of three independent assays. *P<0.05 (ANOVA).

Western blotting was used to analyze the effect of FRAX486 on the expression level of the ABCB1 transporter. ABCB1-overexpressing cells MCF7/ADR were treated with 1 μM FRAX486 for 0, 24, 48, and 72 h. As shown in Figure 2A, the protein level of ABCB1 remained constant throughout the incubation period. These findings implied that FRAX486 had no effect on ABCB1 expression in cancer cells.

### FRAX486 did not affect ABCB1 subcellular localization

**Figure 3:**
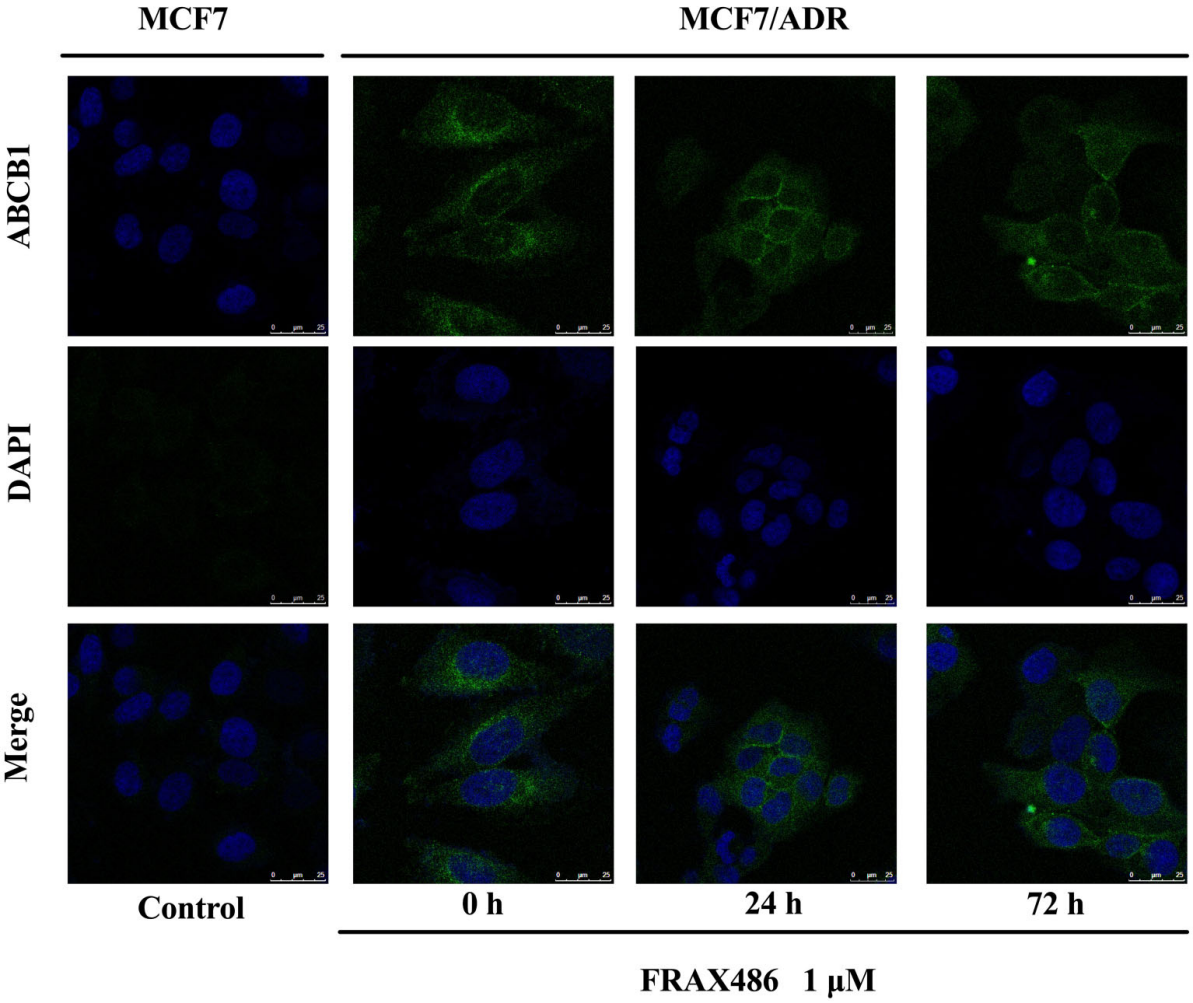
Immunofluorescence images of the effect of FRAX486 on cellular localization of ABCB1 in MCF7/ADR cells (scale bar 25 μm). Green: ABCB1; Blue: nuclei.

Using an immunofluorescence experiment, we investigated the effect of FRAX486 on the subcellular localization of ABCB1 to better understand the mechanism driving multidrug resistance reversal. Figure 3 shows that 1 μM exposure of FRAX486 for different durations had no effect on the subcellular localization of ABCB1. This implied that the FRAX486 reversal effect on multidrug resistance was not mediated by changes in ABCB1 subcellular distribution.

### FRAX486 inhibited the ABCB1 ATPase activity

**Figure 4:**
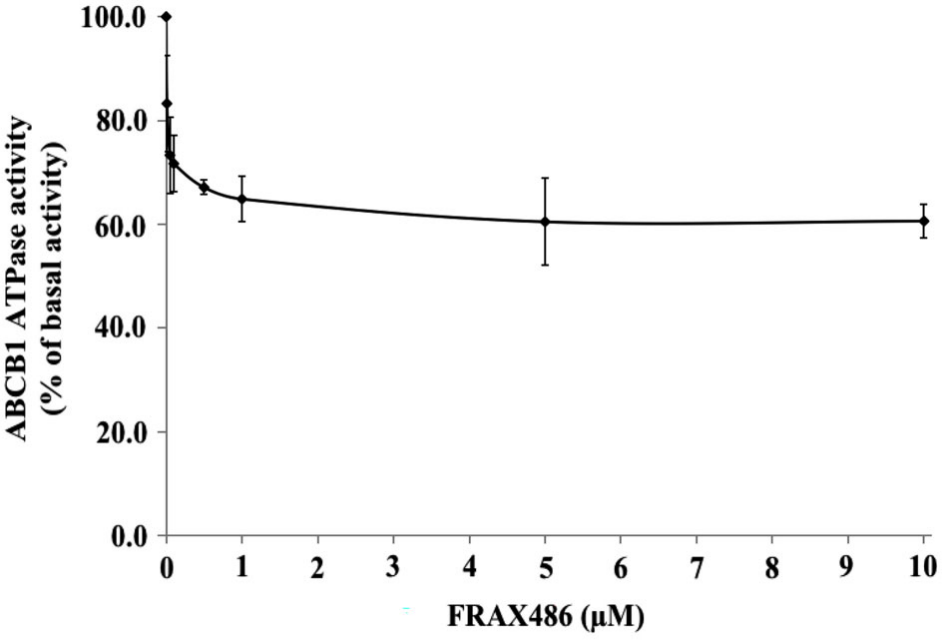
FRAX486 inhibited the activity of ABCB1 ATPase. FRAX486 (0-10 μM) inhibited ABCB1 ATPase activity. Data are reported as means±SD of three independent assays.

In order to conduct a more comprehensive evaluation of the impact of FRAX486 on ABCB1 ATPase activity, we conducted measurements of ATP hydrolysis mediated by ABCB1 in membrane vesicles subsequent to incubation with various concentrations of FRAX486 (ranging from 0-10 μM). FRAX486 effectively suppressed the ABCB1-associated ATPase activity in a dose-dependent manner, reaching a maximum inhibition level of 60.5% compared to the basal activity. The inhibitory effect of FRAX486 reached 50% maximum inhibition (IC_50_) at a concentration of 0.02 μM (Figure 4).

### FRAX486 increased doxorubicin accumulation and reduced efflux within ABCB1-overexpressing cells

**Figure 5:**
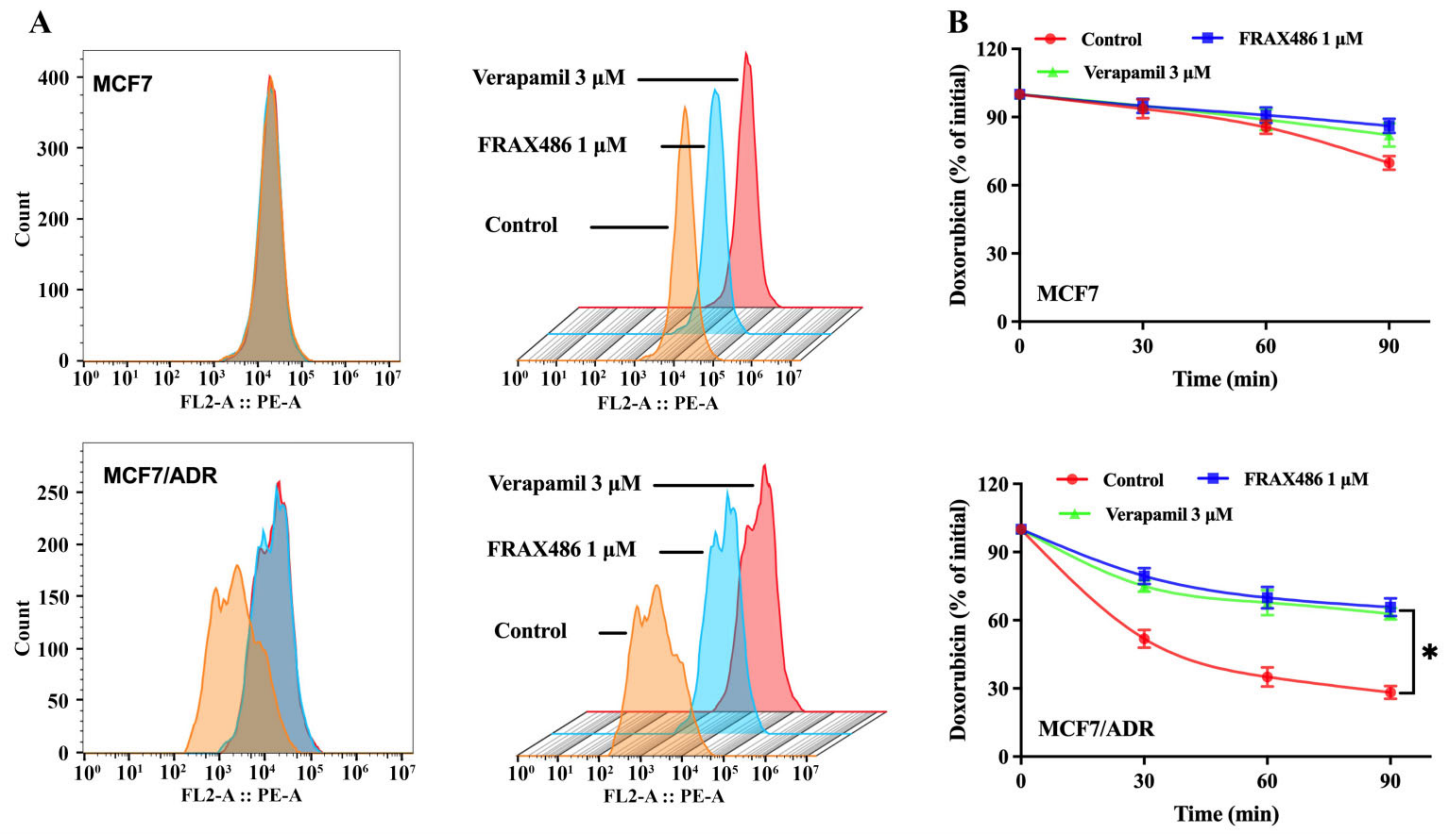
Effect of FRAX486 on intracellular accumulation and efflux of doxorubicin in drug-sensitive and drug-resistant cells. **A**, Accumulation. **B**, Efflux. Data are reported as means±SD. *P<0.05 (ANOVA).

To further investigate the reversal mechanism of FRAX486, a doxorubicin accumulation and efflux assay was conducted. This assay aimed to determine whether FRAX486 could inhibit the substrate efflux function of ABCB1, leading to increased intracellular doxorubicin concentration. FRAX486 increased the intracellular concentration of doxorubicin, specifically in ABCB1-overexpressing MCF7/ADR cells (Figure 5A), and reduced doxorubicin efflux (Figure 5B), while no significant effect was observed in parental cells. These findings suggested that at least a portion of the reversal effect can be attributed to the inhibition of ABCB1 substrate efflux by FRAX486.

### Docking study of FRAX486 with ABCB1 protein model

**Figure 6:**
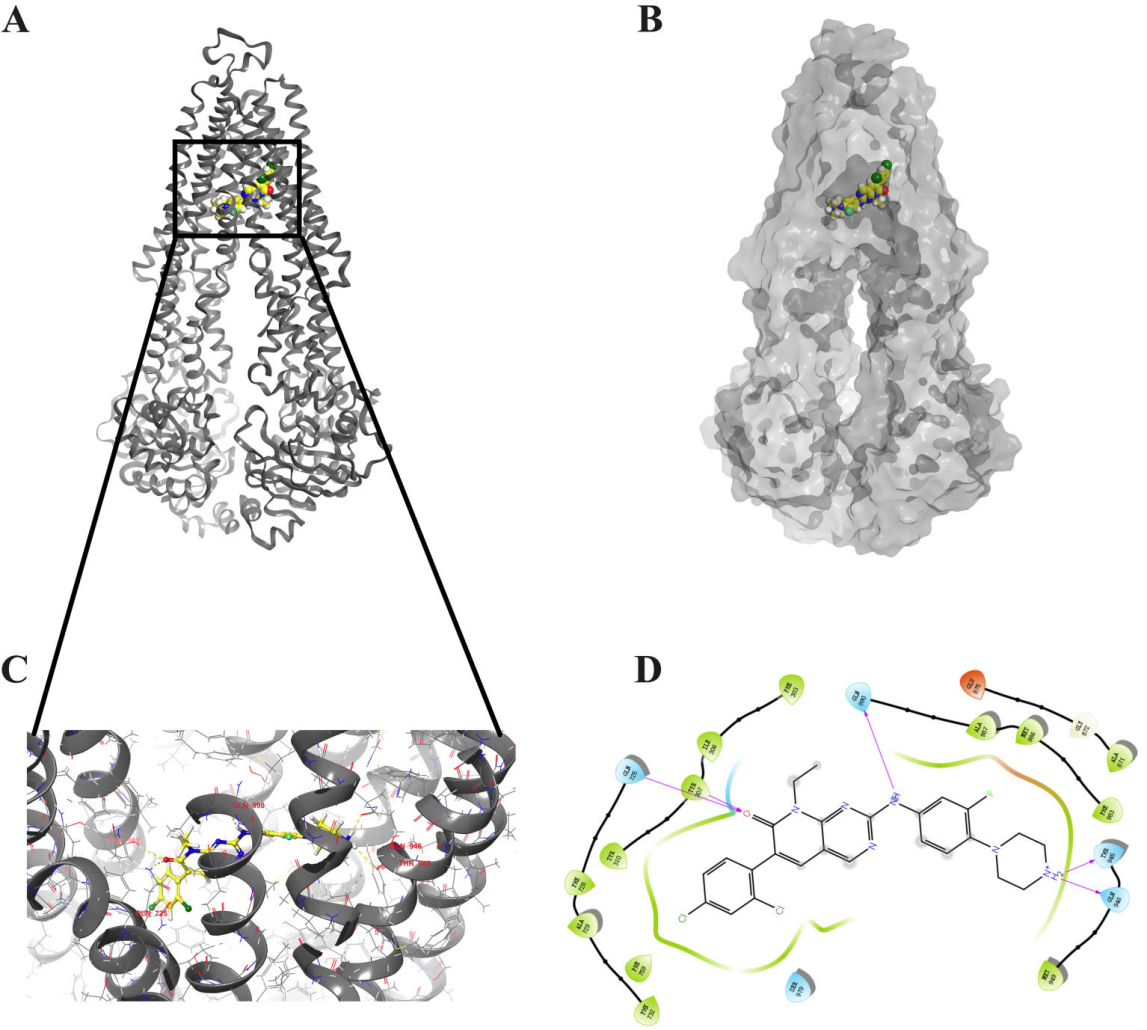
Interaction between FRAX486 and human ABCB1 protein. **A**, Overview of the best-scoring pose of FRAX486 in the drug-binding pocket of ABCB1 protein. ABCB1 is displayed as gray ribbons. FRAX486 is displayed as colored spheres. **B**, Docked complex displayed with protein surface and ligand surface. FRAX486 is displayed as colored spheres. **C**, Details of interactions between FRAX486 and ABCB1 binding pocket. ABCB1 is displayed as gray ribbons. Important residues are displayed as colored bars (gray: carbon; blue: nitrogen; red: oxygen) and FRAX486 in yellow (carbon), blue (nitrogen), and green (chlorine). Hydrogen bonds are displayed as yellow dashed lines. **D**, 2D FRAX486-ABCB1 interaction. Amino acids with 3.0 Å are displayed as colored bubbles - cyan indicates polar residues and green indicates hydrophobic residues. Hydrogen bonds are indicated with purple lines.

To further investigate the ligand-receptor interactions of FRAX486 and ABCB1, we applied docking simulation in the ligand binding site of ABCB1 protein (7A69). FRAX486 docked into the ligand binding site with an affinity score of -9.939 kcal/mol. Details of the ligand-receptor interaction are displayed in Figure 6. The primary factor contributing to the binding of FRAX486 to the ABCB1 protein are the hydrophobic interactions. FRAX486 is positioned and stabilized in the hydrophobic cavity formed by Phe303, Ile306, Tyr307, Tyr310, Phe728, Ala729, Phe732, Ala987, Met986, Phe983, and Met949. Additionally, FRAX486 was further stabilized by hydrogen bonds formed with Tyr307, Gln725, Gln990, Gln946, and Thr945.

## Discussion

MDR in malignant tumors frequently results in the failure of numerous antitumor drugs in clinical settings. Due to their potential function in enhancing the efflux of chemotherapy drugs, the involvement of ATP-binding superfamily members in this process, specifically the ABC transporter, has attracted considerable attention. Both normal tissues and tumor cells express the ABC transporter, but its expression in tumor cells contributes to primary drug resistance (36). ABCB1 was the first ABC transporter to be discovered in humans. Its overexpression is associated with decreased sensitivity to antitumor drug therapies, resulting in poor treatment outcomes and negatively affecting patient survival (37). Therefore, it is necessary to investigate pharmaceuticals that can effectively combat MDR mediated by ABCB1. Drug repurposing has become an attractive strategy in addition to the development of specific ABCB1 regulators.

PAK is a serine/threonine kinase that contributes significantly to the aggressive nature of malignancies. It has been linked to the onset and progression of numerous malignancies, including stomach cancer (38), prostate cancer (39), breast cancer, and thyroid cancer (40), among others. Recent research indicates that PAK is an attractive therapeutic target, and targeting PAK could open up novel avenues for clinical anti-tumor treatments (40).

FRAX486 has shown potential in inhibiting the growth of prostate stromal cells (30). However, it has not been reported whether PAK inhibitors are related to ABC transporters. The findings of the present study revealed that FRAX486 had a reversing effect on drug-resistant cells mediated by ABCB1.

Initially, it was shown *in vitro* that FRAX486 could increase the cytotoxicity of chemotherapy drugs that target ABC transporter substrates in drug-resistant cells with elevated expression of ABCB1. Notably, FRAX486 had no effect on the sensitivity of drug-sensitive cells to chemotherapy drugs or on the sensitivity of drug-resistant cells to non-ABC transporter substrate drugs like cisplatin. These results suggested that FRAX486 has the potential to selectively boost the chemotherapeutic efficacy of drugs transported by ABC transporters in drug-resistant cells.

While previous findings suggest that FRAX486 may reverse ABCB1-mediated MDR, the specific mechanism behind this effect is still not completely explored and requires additional investigation. Western blotting and immunofluorescence assays were performed to further investigate the interaction between FRAX486 and ABC transporters. According to the results, ABCB1 protein expression or cell surface localization were unaffected by FRAX486. These results indicated that the reversal effect of FRAX486 on MDR was not attributable to alterations in the protein expression or cellular distribution of ABCB1. In our ATPase experiments, we made an interesting discovery that FRAX486 demonstrated a dose-dependent reduction in the ATPase activity of ABCB1. The maximal inhibition level reached 60.5%. This finding suggested that one of the mechanisms by which FRAX486 reverses multidrug resistance is by decreasing the energy source for ABCB1 efflux function through the inhibition of ATPase activity. This inhibition can potentially disrupt the normal functioning of ABCB1, leading to enhanced drug accumulation and overcoming drug resistance in cancer cells. To investigate the effect of FRAX486 on drug accumulation and efflux, researchers examined the concentration of the chemotherapeutic agent paclitaxel in resistant cells. The experimental findings revealed that FRAX486 substantially increased paclitaxel accumulation in drug-resistant cells and reduced efflux. Given that ABCB1 is the primary factor contributing to the resistance of MCF7/ADR cells to chemotherapy, FRAX486 may reverse MDR in malignancies by enhancing the intracellular accumulation of chemotherapy drugs by inhibiting the efflux function of ABCB1. Furthermore, molecular docking experiments demonstrated a high affinity between FRAX486 and ABCB1, indicating that FRAX486 inhibits the binding of other substrate medications to ABCB1. These results demonstrated that FRAX486 can bind competitively to the drug-binding sites of the ABCB1 protein, thereby reversing MDR.

### Conclusion

Overall, the findings demonstrated that FRAX486 overcame MDR in cancer via regulating ABCB1 function and enhancing drug accumulation in resistant cells. However, it should be noted that FRAX486 is still under investigation and has not been approved for clinical use by regulatory authorities. To ascertain its safety and efficacy in clinical cancer patients, additional research is required.
